# Supplementary material for: A disassembly-driven mechanism explains F-actin-mediated chromosome transport in starfish oocytes
Source: eLife. 2018 Jan 19;7:e31469. doi: 10.7554/eLife.31469 (PMC5788506; doi:10.7554/eLife.31469)
Supplement: Supplementary file 2. [file elife-31469-supp2.docx]

**Supplementary file 2.** Dimensionless viscoelastic parameters for UtrCH injections

|  | $G_{0}/k_{0}\bar{\eta}_{0}$ | $C{/k}_{0}\bar{\eta}_{0}$ | $\epsilon$ | $\gamma_{\omega}$ | $g$ | m | $\mu$ |
| --- | --- | --- | --- | --- | --- | --- | --- |
| Model M | 0.0561 | 1.0805 | 0.3913 | 3.7600 | 2 | 2 | 1 |
| Model D | 2.1842 | 3.3799 | 0.9993 | 1.4649 | 2 | 1 | 1 |
