# Supplementary material for: A disassembly-driven mechanism explains F-actin-mediated chromosome transport in starfish oocytes
Source: eLife. 2018 Jan 19;7:e31469. doi: 10.7554/eLife.31469 (PMC5788506; doi:10.7554/eLife.31469)
Supplement: Supplementary file 4. [file elife-31469-supp4.docx]

**Supplementary file 4.** Dimensionless viscoelastic parameters for SMIFH2 treatments

|  | $G_{0}/k_{0}\bar{\eta}_{0}$ | $C{/k}_{0}\bar{\eta}_{0}$ | $\epsilon$ | $\gamma_{\omega}$ | $g$ | m | $\mu$ |
| --- | --- | --- | --- | --- | --- | --- | --- |
| Model M | 2.8686 | 1.3007 | 0.5198 | 12.039 | 2 | 2 | 1 |
| Model D | 0.9814 | 0.9625 | 0.3340 | 8.5470 | 1 | 1 | 1 |
